# Supplementary figures and images for: Comparison of gamma and x-ray irradiation for myeloablation and establishment of normal and autoimmune syngeneic bone marrow chimeras
Source: PLoS One. 2021 Mar 17;16(3):e0247501. doi: 10.1371/journal.pone.0247501 (PMC7968675; doi:10.1371/journal.pone.0247501)

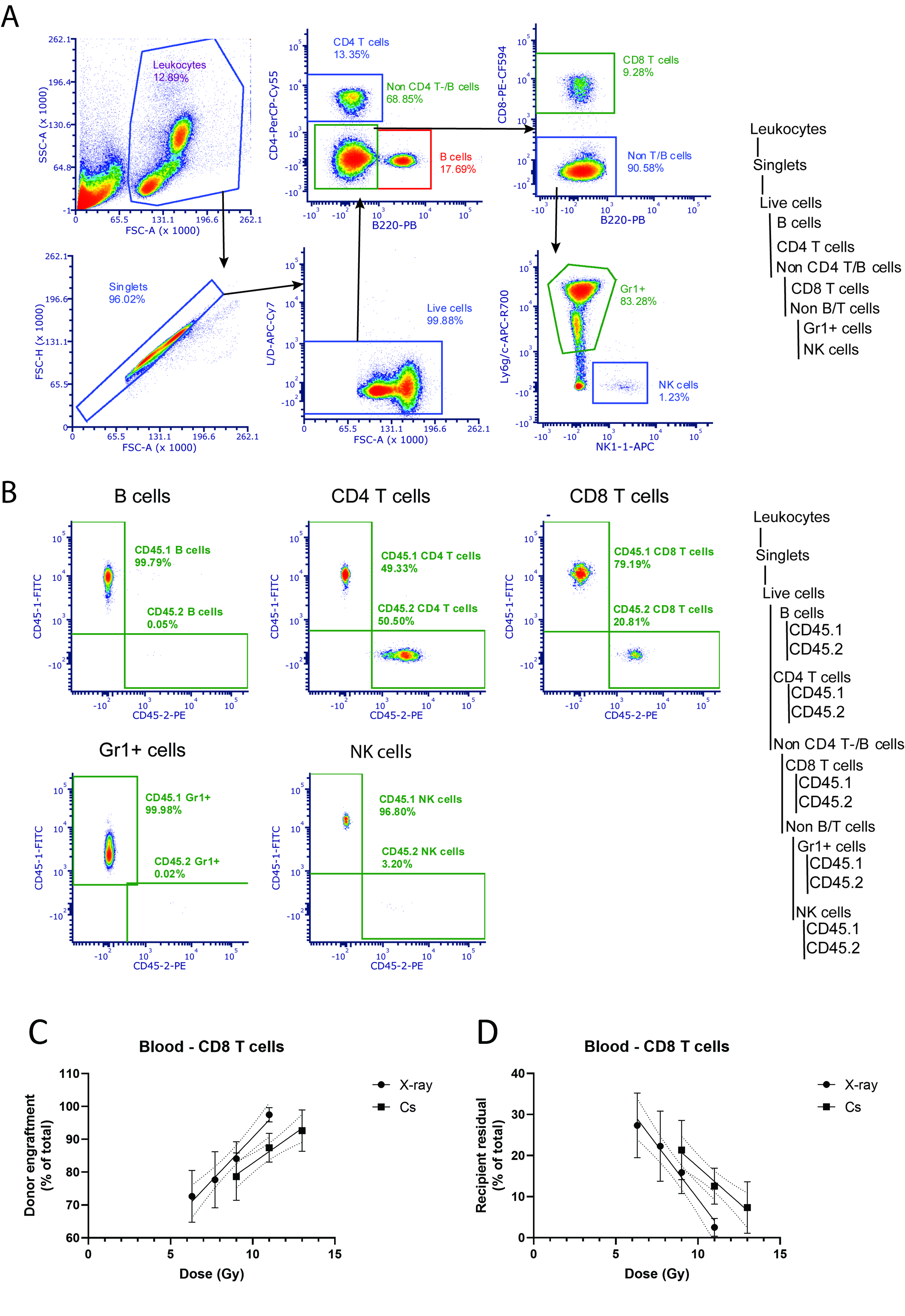

Supplement: S1 Fig — Gating strategy for the investigated immune subsets (A) and chimerism (B). Here the recipient CD45.2 mice received only wild type CD45.1 bone marrow cells. (C) Linear regression plot of donor engraftment (CD45.1 chimerism) as a function of irradiation dose for X-ray and Cs-137 irradiation modalities within the CD8 T cell subset in blood. The dotted lines indicate the 95% confidence interval of the regression lines. (D) Linear regression plot of recipient residual (CD45.2 chimerism) as a function of irradiation dose for X-ray and Cs-137 irradiation modalities within the CD8 T cell subset in blood. The dotted lines indicate the 95% confidence interval of the regression lines. (TIF) [file pone.0247501.s001.tif]

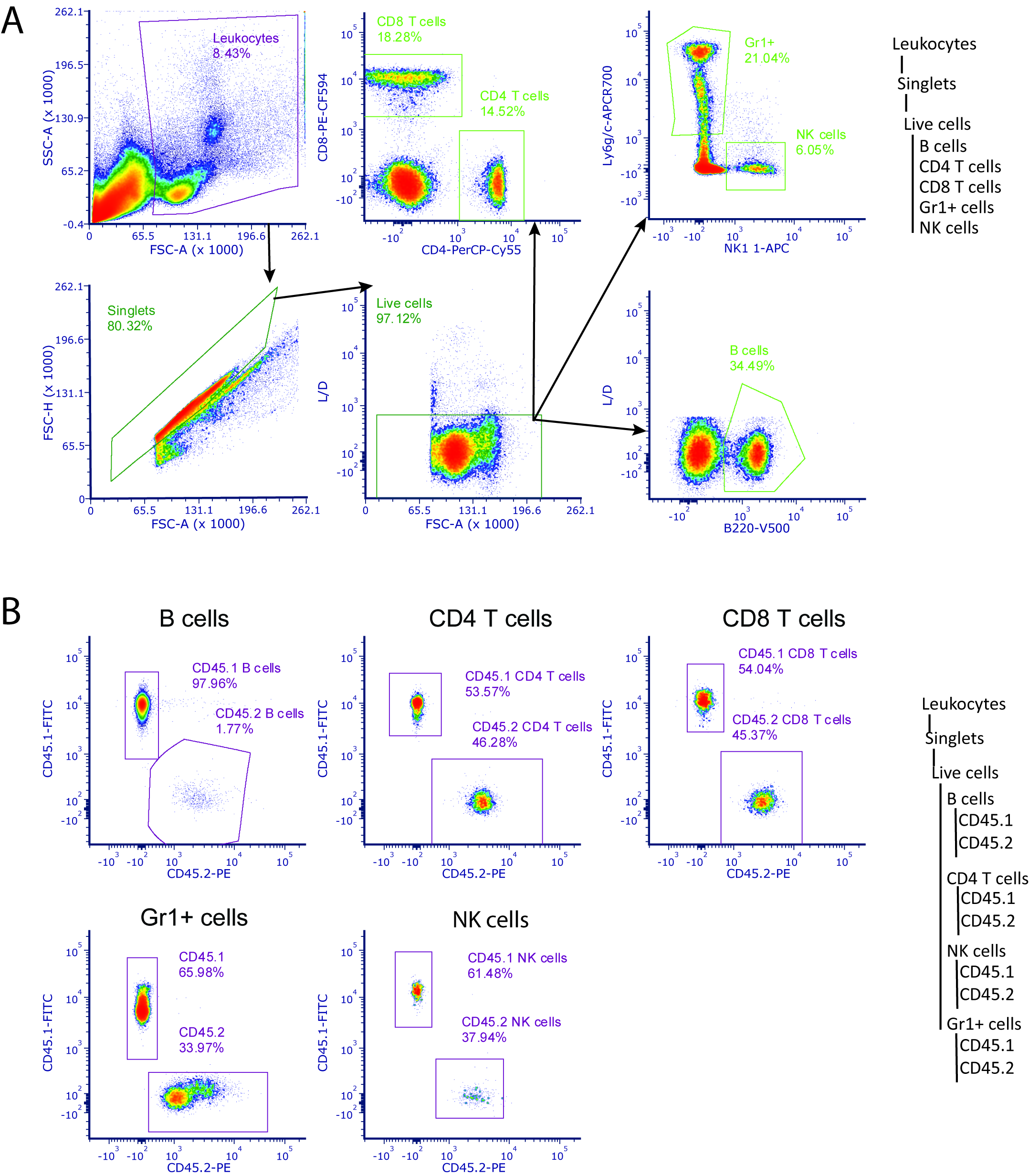

Supplement: S2 Fig — Gating strategy for the investigated immune subsets (A) and chimerism (B). Here the recipient CD45.2 mice received 2 parts wild type CD45.1 cells and 1 part 564Igi bone marrow cells. (TIF) [file pone.0247501.s002.tif]

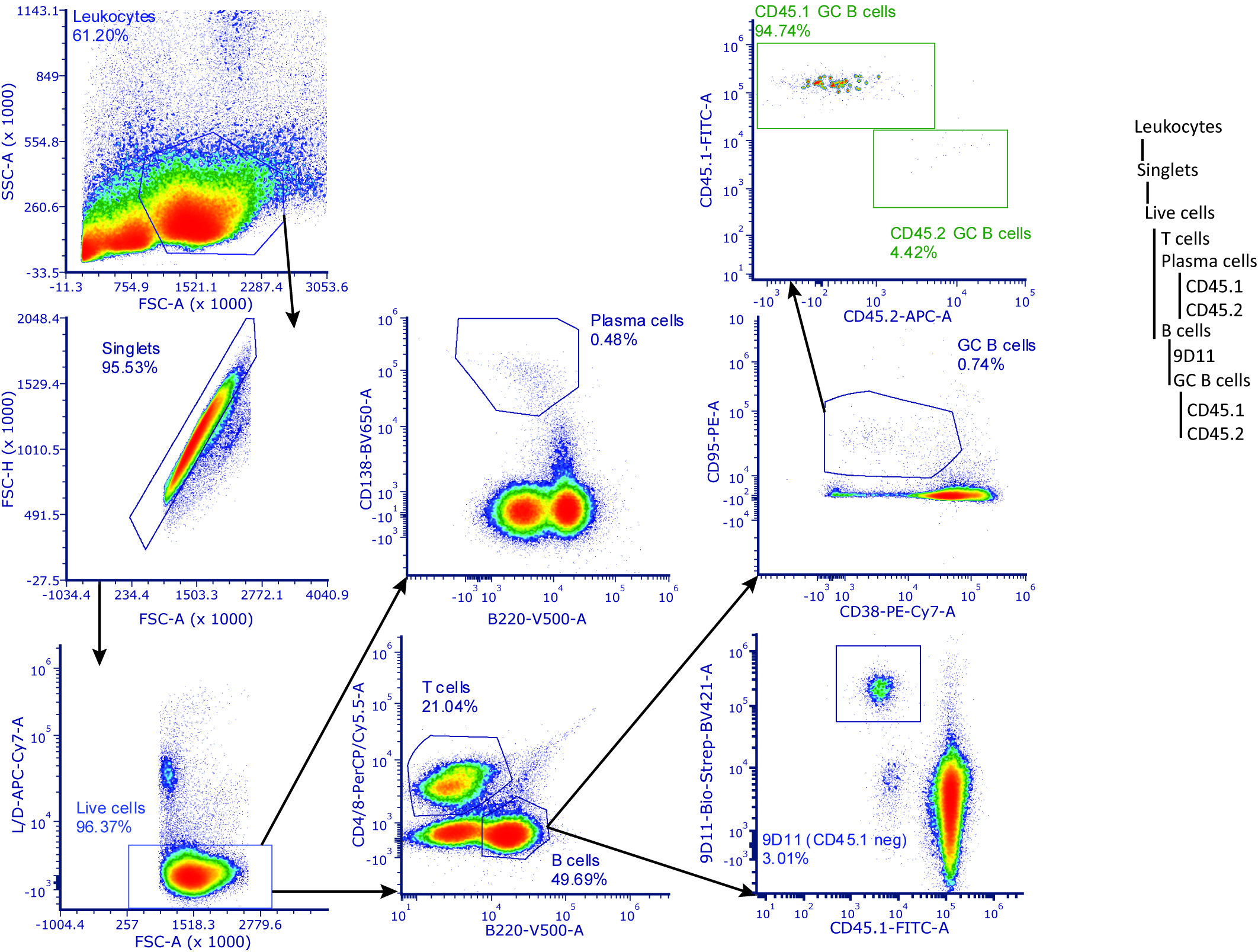

Supplement: S3 Fig — Gating strategy for the investigation of autoimmune phenotype when the recipient CD45.2 mice received 2 parts wild type CD45.1 cells and 1 part 564Igi bone marrow cells. (TIF) [file pone.0247501.s003.tif]
