# Supplementary material for: Comparison of gamma and x-ray irradiation for myeloablation and establishment of normal and autoimmune syngeneic bone marrow chimeras
Source: PLoS One. 2021 Mar 17;16(3):e0247501. doi: 10.1371/journal.pone.0247501 (PMC7968675; doi:10.1371/journal.pone.0247501)
Supplement: Attachment — Submitted filename: Rebuttal to reviewer comments.docx [file pone.0247501.s004.docx]

We would like to start by thanking the Reviewers for their careful evaluation of our manuscript. We realize that these are in many ways challenging times and we deeply appreciate the time taken out of a busy schedule to critically review our work. We were happy to see the overall very positive reception of our manuscript, and fully acknowledge the important issues raised by the Reviewers. Below we have provided a point-by-point response and indicated the changes made in our revised manuscript.

Reviewers' comments:

Reviewer's Responses to Questions

**Comments to the Author**

1. Is the manuscript technically sound, and do the data support the conclusions?

The manuscript must describe a technically sound piece of scientific research with data that supports the conclusions. Experiments must have been conducted rigorously, with appropriate controls, replication, and sample sizes. The conclusions must be drawn appropriately based on the data presented.

Reviewer #1: Yes

Reviewer #2: Yes

2. Has the statistical analysis been performed appropriately and rigorously?

Reviewer #1: Yes

Reviewer #2: Yes

3. Have the authors made all data underlying the findings in their manuscript fully available?

The [PLOS Data policy](http://www.plosone.org/static/policies.action#sharing) requires authors to make all data underlying the findings described in their manuscript fully available without restriction, with rare exception (please refer to the Data Availability Statement in the manuscript PDF file). The data should be provided as part of the manuscript or its supporting information, or deposited to a public repository. For example, in addition to summary statistics, the data points behind means, medians and variance measures should be available. If there are restrictions on publicly sharing data—e.g. participant privacy or use of data from a third party—those must be specified.

Reviewer #1: Yes

Reviewer #2: Yes

4. Is the manuscript presented in an intelligible fashion and written in standard English?

PLOS ONE does not copyedit accepted manuscripts, so the language in submitted articles must be clear, correct, and unambiguous. Any typographical or grammatical errors should be corrected at revision, so please note any specific errors here.

Reviewer #1: Yes

Reviewer #2: Yes

5. Review Comments to the Author

Please use the space provided to explain your answers to the questions above. You may also include additional comments for the author, including concerns about dual publication, research ethics, or publication ethics. (Please upload your review as an attachment if it exceeds 20,000 characters)

Reviewer #1: The manuscript presents a detailed comparison of reconstitution in murine bone marrow transplantation chimeras, using two different sources of irradiation, Cs-137 gamma rays or X-rays, at a series of doses, for myeloablation conditioning. The problem raised by switching to X-ray irradiation is shared by many laboratories worldwide. The study is therefore timely and will be helpful to many researchers depending on irradiation procedures.
Introduction: The theme is very well introduced, clear and well written, with valuable information on the problems linked to Cs-137 and an interesting technical comparison of the two sorts of sources. Experimental conditions are well described.

We thank the reviewer for the positive and constructive critique.

One detail is however missing: it would be helpful to know if special irradiation cages were used to prevent possible burns with X-rays.

We thank the Reviewer for bringing this to our attention, as it is an important consideration for animal welfare and avoiding confounding tissue damage. We used a specially designed X-ray permeable plexiglass pie-cage for high-throughput irradiation. Although this in itself is not designed to avoid burn issues, the design of the irradiator preempts such issues by including a beam collimator that ensures a uniform beam profile and an electrically-operated turntable to ensure uniform dosing. As we outline in the Materials and Methods, we additionally employed a Thoraeus beam conditioning filter, to remove the low keV irradiation, which can otherwise potentially cause superficial tissue burn damage. We have not observed any indication of X-ray burns in any mice included in the study or in our subsequent work. We have included statements to this effect in the Results and Discussion sections.

Results and discussion: Results are provided in two different settings: reconstitution with a complete, syngeneic BM chimeras, and the second one in a mixed chimera model of autoimmune disease. Results are shown clearly, represent a large body of data. Chimerism is assessed with congenic markers in blood, inguinal and mesenteric lymph nodes and spleen. Cytometry gating procedures are well described and adequate.

Data of chimerism in the BM would have been interesting to show, particularly for T cells for which chimerism is more difficult to accomplish.

We concede that this could have been an additional valuable piece of information. Regretfully we did not harvest and analyze bone marrow from the resulting chimeras in the cohorts included in the study. Including this would require a new cohort, and since the Cesium irradiator has now been dismantled, we are unable to follow up with this. However, we feel that the extensive data from blood and lymphoid tissues do give a good indication of the chimerism, also for the T cell compartment, and we hope that the Reviewer will agree that this is acceptable.

Authors conclude that similar chimerism can be obtained with the two different sources of irradiation, allowing future comparisons of generated data. In addition, a similar autoimmune phenotype could be achieved irrespective of the irradiation source. The excess mortality linked to X-ray irradiation, if given at equivalent doses as Cs-137, is a major information, as well as the range of lower doses that can be used to get the same degree of chimerism with X-ray irradiation. Authors conclude clearly that higher conditioning is required to improve T-cell chimerism compared to other cell lineages.

It remains to be discussed why 11 Gy with X-ray source is inducing mortality in the mixed autoimmune chimera model (Fig.4G) but not in the syngeneic model in which 13 Gy, also with X-ray source, is the dose inducing mortality (Fig.2G).

This is a very important point, which we thank the Reviewer for raising. Indeed, it is an astute observation that the syngeneic and mixed chimeras exhibit this difference in sensitivity to X-ray irradiation dose. Since the recipients and donors were very closely age-matched, and had the same gender distribution, and the experiments were conducted under the same conditions, in the same time-frame, with the same instrumentation, with careful dosimetry, and subsequent housing in the same vivarium, we are confident that it is not a technical variation. We hypothesize that the different responses are consequent to the biology of the resulting chimeras. The mixed chimera model exhibits a penetrant autoimmune phenotype, and we suspect that the compound physiological stress of the irradiation/reconstitution and autoimmune phenotype is causative of the higher mortality in this group. We have added a statement to this effect in the Discussion section of the revised manuscript.

Finally, statistical information is only provided on survival curves of Figures 2G and 4G.
Statistical information for the rest of figures should be indicated in the legend figures.

We sincerely apologize for this omission, which we have amended in the revised manuscript. We have indicated throughout that bars and errors represent mean +/- standard error of mean. For analyses of differences in major cell populations presented in Figures 2, 4, and 6, we have employed non-parametric Kruskal-Wallis test (as we could not by-and-large confirm normality within the datasets), and in cases where the Kruskal-Wallis test indicated significant differences (alpha = 0.05) between medians, we have indicated P-values for Dunn’s post-test for those pairwise comparisons which came out significant. For assessment of the relative frequencies of CD45.1 vs. CD45.2 cells presented in Figures 3, 5, and 6, we have employed two-way ANOVA to evaluate irradiation modality as an independent determinant of variation in the datasets, as well as its component role in an interaction effect. As an approach to evaluate the dose dependency statistically and to compare the dose titrations of the two irradiation modalities, we performed linear regression on the donor engraftment (new Supplementary Figure 1C) and recipient residual (new Supplementary Figure 1D) as functions of irradiation dose for CD8 T cells in the blood. This revealed significant differences in dose titration between the two modalities and demonstrated that a lower X-ray dose was required to obtain a similar degree of chimerism. In the revised manuscript, we have indicated statistical approach and significance values in the Figure Legends throughout, and additionally expanded the description of the statistical analyses in the Materials and Methods section.

While valuable variation tendency, in shifting from Cs-137 to X-ray irradiation, have been indicated in this study, authors’ final recommendation is that individual optimization remains necessary.
All criteria that PLOS manuscripts should fulfill are satisfactory. Overall, only minor changes, listed above, are required to accept the manuscript for publication.

Once more, we thank the Reviewer for the constructive critique, which we feel has improved our revised manuscript.

Reviewer #2: Here, the authors compared the effect of two different types of radiations (lethal and total body irradiation) on normal and immunogeneic hematopoietic reconstitution. In this purpose they injected 20 millions of total bone marrow cells from CD45.1 C57B6 mice to CD45.1 recipient mice lethally irradiated either using a Ce137 irradiator or an Xray one. No differences can be observed between Ce137 or Xray irradiators.

Major points

1- The authors used 20 millions of total BM cells for transplantation. With this amount of cells it makes difficult to see any difference after transplantation. Furthermore, recipient mice were sacrificed 43-44 days post transplantation. At this time point, only progenitors are responsible for the progeny observed in the recipient mice. Moreover, long lived cells such as some subsets of T and B cells from the donor mice can still be observed. A longer time point after transplantation should be analyzed, when hematopoietic reconstitution is stable (meaning more than 16 weeks post transplantation). Moreover, to be sure no difference exits between the 2 types of irradiations, some limiting dilution assay should be performed as well.

We appreciate the Reviewer’s point. As we see it, there are three questions at play here: 1) What dose/type of irradiation efficiently ablates the recipient compartment to allow establishment of donor marrow; 2) Does the dose/type of irradiation cause different degrees of inflammation, altering downstream phenotypes; and 3) Does irradiation modality influence long-term HSC behavior. We set out to address, and have addressed, we believe, questions 1 and 2, as stated in the abstract:

“Here, we compared Cesium-137 to 350 keV X-ray irradiation with respect to immune reconstitution, assaying complete, syngeneic BM chimeras and a mixed chimera model of autoimmune disease.”

We feel that question 3 is beyond the scope of the present work, and would be better suited for a focused HSC study. As we work within the field of immunology, we feel that others are better poised to tackle such a question pertaining to the biology of HSCs. We note that 6-8 weeks rest period post-reconstitution is a standard approach in the literature for partial and complete chimeras alike, for use in immunological studies. For a few recent examples, see e.g. the following examples of our own work and that of others (Degn et al., 2017; Jing et al., 2020; Müller-Winkler et al., 2021; Yeh et al., 2018). Thus, while we appreciate that the experimental approaches are different in the HSC field, we feel that the present work serves the intended purpose of reassuring researchers working in immunology and related fields that they can successfully transition to X-ray irradiators with some optimization.

Specifically for the mixed chimera model, we have previously performed analyses of the kinetics of reconstitution. To this end, we generated mixed bone marrow chimeras:

- 1 part CD45.2 564 and 2 parts CD45.1 WT
- 1 part CD45.2 564K- and 2 parts CD45.1 WT OR 1 part CD45.2 WT and 2 parts CD45.1 WT

then analyzed at weeks 2, 3, and 4, and pooled this with previous data from week 6.

Kinetics curves for splenic B220, Idiotype and GC frequencies are presented below.

Mean +/- SEM for from 1-7 mice per time point, with an average of 4.6 mice per group per time point.

We observed a bulge of B cells (B220) following initial reconstitution, dropping to normal levels (indicated by gray normal area as measured in 7 B6 mice) at 3-4 weeks post reconstitution. There was an even more marked and dramatic bulge of Id+ cells following reconstitution of 564 mixed chimeras, also ‘normalizing’ at 3-4 weeks. The baseline of Id+ cells was constant in B6 and K- mixed chimeras. The splenic GC frequency remained low until at least 4 weeks, but increased dramatically by 6 weeks in 564 mixed chimeras, but not in controls.

Our interpretation is that the irradiation empties the normal compartments and ablates GC. There is an initial repopulation by 564-derived B cells, which may emerge more rapidly from the BM than their wt counterparts as they do not need to undergo recombination. The 564 B cells are allowed to populate the periphery because of relaxed negative selection, as negative selection is dependent on the presence of normal competitor cells (Schmidt and Cyster, 1999). As the compartment is filled with normal wt B cells, negative selection at the transitional stage is reestablished, leading to a drop in naïve 564 B cell frequency in the periphery. At this point, we surmise that tolerance has already been broken at the (pre-)GC (potentially T cell) level, but the GC phenotype does not manifest until sometime between 4 and 6 weeks.

Hence we perceive 6 weeks in this model to be the earliest robust time-point for analyses, and irrespective of potential subtle, long-term changes in various hematopoietic compartments, we believe our conclusion stands, i.e., that X-ray irradiation is a viable alternative conditioning method in the described models.

2- It is not very clear how many times experiments were done and the quality of each figure is very low rending difficult to read them.

The syngeneic chimera experiment presented here was performed twice in the parallel setup, i.e., where cohorts were split and irradiated with dose titration on the two instruments. The mixed chimera experiment was performed once in the parallel setup. This is already stated in the Materials and Methods in the section “Generation of BM chimeras”:

“In two experiments, recipients were reconstituted with only CD45.1 BM cells, whereas in the third they received BM cells from 564Igi donors and from CD45.1 donors at a ratio of 1:2.”

It was not possible to perform more than three rounds of experiments in the ‘overlap period’, where we had both irradiators available at our institution and hence could run experiments in parallel. However, at our facility, we have performed 19 rounds of chimeras using the Cesium irradiator *before* the transition, and 30 rounds of chimeras using the X-ray irradiator *after* the transition, using the respective optimized doses, and these experiments have confirmed that the optimized doses we arrived at for syngeneic and mixed chimeras yield robust reproducibility.

Regarding the quality of the figures, we apologize for the low quality of rendering, which is due to a down-sampling in the manuscript draft in the submission system. The original figures are high-resolution and can be accessed by clicking the link on the top-right-hand corner of each figure in the PDF.

In conclusion, once more, we would like to thank the Reviewers for their constructive critique, which we feel has significantly improved our revised manuscript.

Finally, we would like to thank the Editor for handling our submission.

**References included in this rebuttal:**

Degn, S.E., van der Poel, C., Firl, D.J., Ayoglu, B., Qureshah, Al, F.A., Bajic, G., Mesin, L., Reynaud, C.-A., Weill, J.-C., Utz, P.J., Victora, G.D., Carroll, M.C., 2017. Clonal Evolution of Autoreactive Germinal Centers. Cell 170, 913–926. doi:http://dx.doi.org/10.1016/ j.cell.2017.07.026

Jing, Y., Dai, X., Yang, L., Kang, D., Jiang, P., Li, N., Cheng, J., Li, J., Miller, H., Ren, B., Gong, Q., Yin, W., Liu, Z., Mattila, P.K., Ning, Q., Sun, J., Yu, B., Liu, C., 2020. STING couples with PI3K to regulate actin reorganization during BCR activation. Sci Adv 6, eaax9455. doi:10.1126/sciadv.aax9455

Müller-Winkler, J., Mitter, R., Rappe, J.C.F., Vanes, L., Schweighoffer, E., Mohammadi, H., Wack, A., Tybulewicz, V.L.J., 2021. Critical requirement for BCR, BAFF, and BAFFR in memory B cell survival. Journal of Experimental Medicine 218. doi:10.1084/jem.20191393

Schmidt, K.N., Cyster, J.G., 1999. Follicular exclusion and rapid elimination of hen egg lysozyme autoantigen-binding B cells are dependent on competitor B cells, but not on T cells. J. Immunol. 162, 284–291.

Yeh, C.-H., Nojima, T., Kuraoka, M., Kelsoe, G., 2018. Germinal center entry not selection of B cells is controlled by peptide-MHCII complex density. Nat Commun 9, 928. doi:10.1038/s41467-018-03382-x
